# Enhanced but hypofunctional osteoclastogenesis in an autosomal dominant osteopetrosis type II case carrying a c.1856C>T mutation in *CLCN7*

**DOI:** 10.1038/boneres.2016.35

**Published:** 2016-11-29

**Authors:** Xiang Chen, Kun Zhang, Janet Hock, Chunyu Wang, Xijie Yu

**Affiliations:** 1 Laboratory of Endocrinology and Metabolism, Department of Endocrinology, State Key Laboratory of Biotherapy, West China Hospital, Sichuan University, Chengdu, China; 2 The Polis Center, Indiana University-Purdue University Indianapolis, Indianapolis, IN, USA

## Abstract

Type II autosomal dominant osteopetrosis (ADO2), which is the most common form of osteopetrosis, is caused by heterozygous mutations in the chloride channel 7 (*CLCN7*) gene. The osteopetrosis of ADO2 has been attributed to hypofunctional osteoclasts. The mechanism underlying the abnormality in osteoclast function remains largely unknown. This study was designed to investigate gene mutations and osteoclast function in a case that was clinically diagnosed as ADO2. Genomic DNA was extracted from blood samples of this patient, and the 25 exons of *CLCN7* were amplified. Peripheral blood from the ADO2 subject and a healthy age- and sex-matched control was used to evaluate osteoclastogenesis, osteoclast morphology, and bone resorption. Analysis of DNA from the patient showed a germline heterozygous missense mutation, c.1856C>T (p.P619L), in exon 20 of *CLCN7*. A similar homozygous mutation at this site was previously reported in a patient with autosomal recessive osteopetrosis. When cultured, the peripheral blood mononuclear cells (PBMCs) from the ADO2 patient spontaneously differentiated into mature osteoclasts *in vitro*. The ADO2 patient’s PBMCs formed enhanced, but heterogeneous, osteoclasts in both the presence and absence of macrophage-colony stimulating factor, and nuclear factor-ĸB ligand. Bone resorption was reduced in the ADO2 patient’s osteoclasts, which exhibited aberrant morphology and abnormal distribution of integrin a_v_β_3_. Gene analysis found increased *c-fos* expression and reduced *RhoA* and *integrin beta 3* expression in ADO2 cells. In conclusion, our data suggest that enhanced, heterogeneous osteoclast induction may be an intrinsic characteristic of ADO2.

## Introduction

Osteopetrosis is a rare genetic bone disorder. There are three clinical groups of osteopetrosis: autosomal recessive osteopetrosis, which is fatal during the early childhood; intermediate osteopetrosis, which appears during the first decade of life but does not develop a malignant course; and adult-onset autosomal dominant osteopetrosis (ADO), in which patients present mainly with bone-related symptoms and have full life expectancy. Adult-onset ADO is further divided into type I ADO and type I ADO.^1^ Increased thickness of the cranial vault is a typical finding in type I ADO, whereas end-plate thickening of the vertebrae and endobones in the pelvis are typical imaging features in type II ADO.^1^

Autosomal dominant osteopetrosis type II (ADO2), the most common form of osteopetrosis, is caused by heterozygous mutations in the chloride channel 7 (*CLCN7*) gene, which is located on chromosome 16p13.3.^2–6^A recent study confirmed that CLCN7 is a Cl^−^/H^+^antiporter with a 2:1 stoichiometry.^7,8^ Affected individuals show diffuse osteosclerosis, which is manifested as bone-in-bone and “sandwich vertebrae”, as well as elevated levels of tartrate-resistant acid phosphatase (TRAP) and the BB isoform of creatine kinase (CK-BB) in serum.^2,9^ Currently, it is thought that in ADO2, reduced bone resorption resulting from defective osteoclast (OC) functionality leads to the clinical and radiographic findings of ADO2 osteopetrosis. However, the mechanism underlying the osteoclast dysfunction that leads to reduced bone resorption remains to be elucidated.

Another puzzle in studying the bone etiology of ADO2 is the variability in clinical phenotypes.^10,11^ The penetrance of ADO2 is ~66%,^2^ and ~33% of all carriers with ADO2 gene mutations are asymptomatic and have normal radiographs.^8,12^ Some ADO2 gene mutation carriers exhibit bone abnormalities in radiographs but lack overt clinical symptoms, whereas others may present clinically with multiple fractures, osteomyelitis, and cranial nerve deficits.^10,13^ It is speculated that background modifier genes may influence the penetrance of the *CLCN7* gene.^8^

ADO2 is currently incurable, unlike autosomal recessive osteopetrosis (ARO), which is a more severe form of osteopetrosis that may be treated by bone marrow transplantation. Identifying the mechanisms underlying ADO2 is critical for the development of effective therapies for treating this form of osteopetrosis.

The chloride channel CLCN7 has been considered as a potential new drug target for osteoporosis.^14,15^ Currently, the drugs available to treat osteoporosis are limited to anti-resorptives or anabolic agents. Bisphosphonates, which form the first-line anti-resorptive drug class used to treat osteoporosis, concurrently inhibit osteoblast (OB) and OC functions.^16^ However, OC and OB functions seem to be uncoupled in ADO2 because OB function remains normal despite impaired OC function.^8^ Previous studies have reported enhanced OC formation in ADO2 bone marrow, suggesting the compensatory enhanced proliferation of OCs in response to reduced bone resorption.^17^ ADO2 OCs represent an ideal cell model with which to investigate the auto-regulation of osteoclastogenesis and the interactions between OCs and OBs.

Here, we report an ADO2 patient carrying the c.1856C>T (p.P619L) mutation in the *CLCN7* gene. An *in vitro* study of OCs induced from his peripheral blood mononuclear cells (PBMCs) showed enhanced but hypofunctional osteoclastogenesis.

## Materials and methods

### Patient

The research protocol was approved by the ethics committee of West China Hospital of Sichuan University. All participants provided written informed consent before participating in the study.

**Figure 1:**
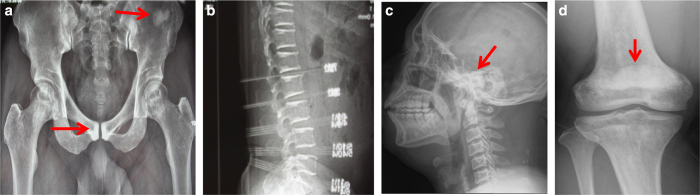
Radiographic examination reveals diffuse osteosclerosis in an ADO2 patient. (**a**) Bone-in-bone in the pelvis wing (arrow). High-bone density under the cartilage in the pubic symphysis (arrow). (**b**) ADO2-characteristic sandwich shape of the vertebral body. (**c**) Osteosclerosis in the base of the skull (arrow). (**d**) Osteosclerosis in the distal metaphysis of the femur (arrow).

A 43-year-old man was first seen at the outpatient unit of West China Hospital, with complaints of discomfort in his lower extremities for 5–6 years. The radiographs revealed “bone-in-bone” in the pelvis wing, a “sandwich” spine, and sclerosis of the base of the skull, which are characteristic of ADO2 (Figure 1). The patient’s serum TRAP-5b was significantly elevated (>10 U·L^−1^), and his parathyroid hormone (PTH) level was at the upper limit of the normal range (6.18 pmol·L^−1^).The patient’s type I collagen C-telopeptide (CTX) level (0.364 ng·mL^−1^) was at the lower limit of the normal range. The patient’s serum levels of bone-specific alkaline phosphatase (B-ALP), 25-OH-VD, calcium, phosphate, and magnesium were within the normal range (Table 1). The patient’s mean bone mineral density, *T* and *Z* scores of lumbar vertebrae, L1–L4, were 1.65, 4.7, and 4.5 g·cm^−2^, respectively. His mean bone mineral density, *T* and *Z* scores for the femoral neck were 1.292, 2.4, and 2.5 g·cm^−2^, respectively. The patient had no history of fracture. According to the clinical features and imaging data of the patient, he was clinically diagnosed as ADO2, and other types of osteopetrosis and skeletal fluorosis were excluded. No similar symptoms were found in his first-degree relatives.

### Materials

α-MEM and fetal bovine serum were obtained from GIBCO (Gibco Europe, Uxbridge, UK). Ficoll-Hypaque solution was obtained from Hao Yang Bio. (Tianjing, China). Recombinant human macrophage-colony stimulating factor (M-CSF) and nuclear factor-ĸB ligand (RANKL) were purchased from R & D Systems (Minneapolis, MN, USA). Anti-human RAC1, vinculin, β-actin, and integrin a_v_β_3_ antibodies were obtained from Abcam (Cambridge, MA, USA). Alexa Fluor 488 goat anti-mouse IgG antibody was obtained from Thermo Fisher Scientific (Waltham, MA, USA). The TRAP staining kit was purchased from Sigma-Aldrich (St. Louis, MO, USA). Rhodamine-conjugated phylloidin for labeling of filamentous actin was obtained from Sigma-Aldrich. Total mRNA was extracted using Trizol reagent (Invitrogen, Waltham, MA, USA). The reagents for reverse transcription and quantitative fluorescence reaction were purchased from TAKARA (Dalian, China).

### Mutation analysis

Genomic DNA was extracted from blood samples of the ADO2 patient, as well as his daughter and son. The DNA sequence of the *CLCN7* gene was obtained from an online database (NC_000016). Primers were designed to amplify fragments containing exons and the exon/intron junctional sequences, using Primer-BLAST (primer sequences available on request). A total of 25 exons of the *CLCN7* gene were amplified by polymerase chain reaction and were directly sequenced.

### Cell culture

A total of 15 mL of heparin anti-coagulated peripheral blood was obtained by venipuncture from the patient, and a healthy age- and sex-matched control. PBMCs were isolated using Ficoll-Hypaque solution. The cells were washed in phosphate-buffered solution (PBS) twice, and plated on 24-well plates at a density of 1×10^6^ per well at 37 °C in α-MEM, supplemented with 10% FBS, 1% penicillin/streptomycin and 25 ng·mL^−1^ of M-CSF. Non-adherent cells were removed by a medium change at 48 h, and every 3 days thereafter. OC differentiation was induced 6 days later, with medium supplemented with both 25 ng·mL^−1^ of M-CSF and 30 ng·mL^−1^ RANKL. PBMCs in three wells from each subject were cultured in the absence of M-CSF and RANKL as a negative control.

### Osteoclast formation assay

TRAP staining was performed after the cells had been cultured in M-CSF and RANKL for 7 days. The cells were fixed in 3% (wt/vol) formaldehyde at room temperature, and stained according to a protocol provided by Sigma-Aldrich. TRAP-positive cells containing three or more nuclei were counted as OCs, using bright-field light microscopy.

### Bone resorption

At the beginning of OC differentiation, 6 mm×6 mm bovine cortical bone slices were put into cell culture wells. At 7 days after co-culture, the slices were removed and evaluated for OC morphology and pit formation by scanning electron microscopy (INCA PENTAFET X3, Oxford Instruments, Abingdon, Oxfordshire, UK). Half of the slices were fixed in 2.5% PBS-buffered gluteraldehyde for 2 h at 4 °C, followed by gradient alcohol dehydration to remove water content. The remaining slices were assessed for lacunar resorption after the ultrasonic removal of adherent cells.

### Quantitative real-time PCR of OC-related genes

At 7 days after the induction of differentiation of OC, total RNA was extracted with Trizol reagent. cDNA was synthesized from 1 μg RNA with PrimeScript RT reagent kit. Gene expression levels were evaluated by real-time PCR, using SYBR Premix Ex *Taq*II, in a volume of 25 μL containing 12.5 μL Premix Ex *Taq*II, 1μL forward primer, 1 μL reverse primer, 8.5 μL dH_2_O, and 2μL cDNA. The reaction conditions included an initial denaturation step at 95 °C for 30 s, followed by 40 cycles of 95 °C 5 s→60 °C 30 s. The relative expression levels of known OC-related genes were normalized to GAPDH and analyzed using the 2^−^^△△Ct^ method. The sequence of oligonucleotide primers is available on request.

### Fluorescence labeling of cytoskeleton-related proteins

Cells were fixed in 4% PBS-buffered paraformaldehyde at room temperature for 10 min, washed twice in PBS, and then permeabilized with 0.5% Triton X-100 in PBS for 5 min at room temperature. The cells were incubated with the primary antibody (diluted 1:100) for 1 h at room temperature, followed by fluorescein-conjugated anti-mouse secondary antibodies (diluted 1:500) for an additional 30 min at room temperature. Microfilaments were labeled by incubating the cells with 10 mg·mL^−1^ rhodamine-conjugated phalloidin. The staining was evaluated using a Zeiss Observer D1 microscope (Zeiss, Oberkochen, Germany).

## Results

### c.1856C>T (p. P619L) mutation in *CLCN7*

Analysis of DNA from the ADO2 patient revealed a germline heterozygous missense mutation, c.1856C>T, in exon 20 of *CLCN7* (HGNC: 2025; OMIM: 602727) (Supplementary Figure 1). This mutation caused the 619th amino acid proline (P) to be changed to leucine (L). This mutation was not present in DNA from his daughter and son. This mutation has not been previously reported in ADO2 patients. Interestingly, the homozygous mutation of c.1856C>T (p.P619L) was reported in a case with ARO.^18^

### Enhanced OC formation in the ADO2 patient

**Figure 2:**
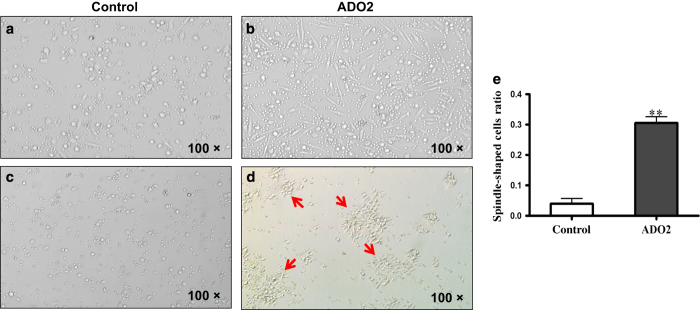
Hyperproliferation of peripheral blood mononuclear cells (PBMCs) from the type II autosomal dominant osteopetrosis (ADO2) patient. (**a**,**b**) Proliferating cells were cultured in the presence of macrophage-colony stimulating factor (M-CSF). More spindle-shaped cells were formed in PMBCs from the ADO2 patient (**b**) than from the control. (**a**,**c**,**d**) Cells were cultured in the absence of M-CSF. The control cells maintained their original circular shape. (**c**) ADO2 cells formed large numbers of spindle-shaped cells in colonies (arrows). (**d**,**e**) The ratio of spindle-shaped cells to total cells was significantly increased in the culture from the ADO2 patient.

Cells were cultured in the presence of M-CSF to induce macrophage expansion over 6 days of culture. Although the same number of cells was seeded from the ADO2 and control samples, the ratio of spindle-shaped cells to total cells in a field was significantly increased in the cultures from the ADO2 patient (30.5 vs 3.95%, *P*<0.01) (Figure 2a,b and e).

Interestingly, the ADO2 and control cells exhibited markedly different morphology when cultured in the absence of M-CSF (Figure 2c and d). Control cells mostly maintained their original circular shape (Figure 2c), whereas the ADO2 cells formed large numbers of spindle-shaped cells, which aggregated together to generate many colonies (Figure 2d). These results indicate that ADO2 osteoclastic progenitor cells show a strong proliferative ability.

**Figure 3:**
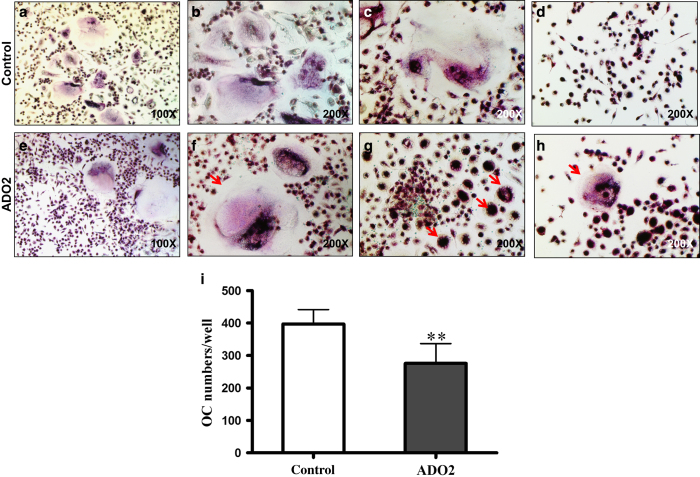
Enhanced but heterogeneous osteoclast (OC) formation in autosomal dominant osteopetrosis type II (ADO2). Peripheral blood mononuclear cells (PBMCs) were induced to differentiate into OCs in the presence of macrophage-colony stimulating factor (M-CSF) and RANKL for 7 days (**a**–**c**,**e-g**). (**a**–**c**) TRAP-positive multinuclear cells in the control. Note the heterogeneous OCs in the ADO2 cells (**e**-**g**). (**e**) Seemingly normal OCs. (**f**) Giant OC with large numbers of nuclei (arrow). (**g**) Abnormal TRAP-positive multinuclear cells (arrows). The ADO2 osteoclast nuclei were always arranged in circles. (**d**,**h**) PBMCs were cultured in the absence of M-CSF and RANKL for 7 days. TRAP-positive multinuclear cells spontaneously formed from ADO2 cells (red arrows) (**h**). No multinucleated osteoclast-like cells were observed in the control cells (**d**). (**i**) The number of seemly normal OC in the ADO2 culture was significantly lower than that in the control culture.

Next, osteoclastic progenitor cells from the ADO2 case and control were induced to differentiate by adding M-CSF and RANKL after 6 days of culture in the proliferation medium. The cells were fixed and stained for TRAP to identify osteoclasts with 3 or more nuclei. The cell types and their morphology were extraordinarily heterogeneous in ADO2, which made it difficult to calculate the OC numbers and the nucleus numbers/OC. Although some OCs exhibited morphology similar to control OCs (Figure 3e), others appeared as giant-sized OCs with a large number of nuclei (Figure 3f). The majority of the OCs exhibited aberrant morphology (Figure 3g). Aberrant OC-like cells stained strongly for TRAP, aggregated together and exhibited a circular arrangement of their multiple nuclei (Figure 3g). OC numbers in 6 wells from control and ADO2 subjects were counted. In ADO2, only seemingly normal OCs were counted, and the average OC numbers/well were significantly reduced in ADO2 (276±61 vs 397±45, *P*<0.01, Figure 3i). In the control cells, the average number of nuclei per cell was 4.72±1.90.

Some TRAP-positive multinucleated cells spontaneously formed from ADO2 cells when cultured in the absence of M-CSF and RANKL (Figure 3h). No multinucleated osteoclast-like cells were observed in the control cells under the same culture conditions (Figure 3d). Of interest, the aggregated cells in ADO2 were all strongly positive for TRAP staining, indicating these cells might be osteoclastic progenitors (Figure 3h). These data suggested that ADO2 PBMCs have a strong ability to proliferate and differentiate into OCs.

### Abnormal morphology and bone resorption in ADO2 OCs

**Figure 4:**
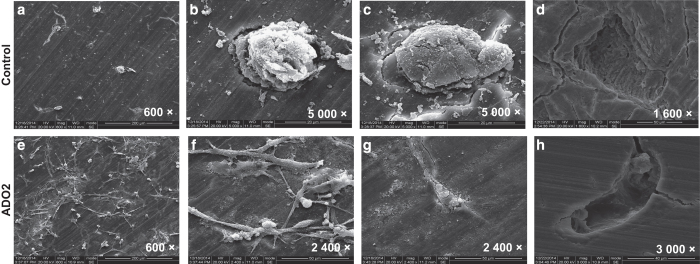
Abnormal morphology and bone resorption in type II autosomal dominant osteopetrosis (ADO2) osteoclasts (OCs). Scanning electron microscope photomicrographs of cell morphology and pit formation. (**a**–**c**) Adherent cells from the control OC cultures. (**d**) Lacunar resorption after ultrasonic removal of the adherent cells in the control OC cultures. (**e**,**f**) Many adherent cells were observed in the ADO2 OC cultures. The adherent cells were irregularly spindle or stellate shaped (**e**-**g**). (**h**) Shallow lacunar resorption after ultrasonic removal of the adherent cells in the ADO2 OC culture.

OCs were co-cultured with bone sections to observe changes in OC morphology and bone resorption. In the control cell co-cultures, the few, scattered adherent cells on the bone sections were cauliflower-like, with multiple protrusions and wrinkles, and were located at sites of bone resorption (Figure 4a–c). By contrast, in ADO2 cell co-cultures, there was a marked increase in adherent cells, and these cells were all irregularly spindle or satellite-shaped, without obvious protrusions and wrinkles. Most of these cells did not induce bone resorption. Only a few irregularly spindle-shaped cells were associated with bone surfaces (Figures 4e–g), and their resorption lacunae were shallower and smaller than those produced by the control OCs (Figure 4d and h).

### Different gene expression profiles of OC-related genes in ADO2 OCs

**Figure 5:**
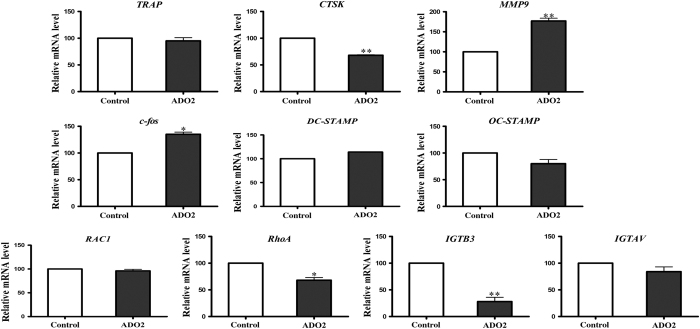
Expression profiles of OC-related genes. Compared with control OCs, the expression levels of *TRAP*, *RAC1*, *DC-STAMP*, *OC-STAMP*, and *ITGAV* in ADO2 cells were similar. However, the expression levels of *CTSK*, *RhoA,* and *ITGB3* in ADO2 cells were significantly down-regulated, whereas the expression levels of *MMP9* and *c-fos* were significantly increased in ADO2 cells compared with control cells.

ADO2 OCs presented with an enhanced proliferation and differentiation ability, as well as abnormal cellular morphology.The expression levels of OCs-related genes encoding several proteolytic enzymes, including *TRAP*, *cathepsin K* (*CTSK*), and *matrix metallopeptidase 9* (*MMP9*), OC differentiation (*c-fos*), OC fusion (*DC-STAMP*, *dendritic cell-specific transmembrane protein*; *OC-STAMP*, *osteoclast stimulatory transmembrane protein*), and cytoskeleton-related proteins, including *RAC1, RhoA*, *integrin alpha V* (*ITGAV*), *integrin beta 3* (*ITGB3*) were measured. Compared with control OCs, the expression levels of *TRAP*, *RAC1*, *DC-STAMP*, *OC-STAMP*, and *ITGAV* in ADO2 cells were similar. However, the expression levels of *CTSK*, *RhoA*, and *ITGB3* were significantly down-regulated in ADO2 cells. In addition, the expression levels of *MMP9* and *c-fos* were significantly increased in ADO2 cells (Figure 5).

### Abnormal distribution of integrin a_v_β_3_ in ADO2 OCs

**Figure 6:**
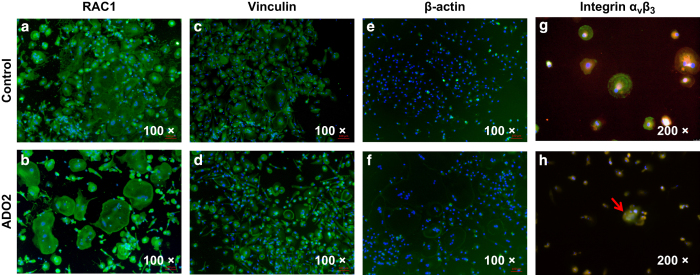
Immunofluorescence staining for cytoskeleton-related proteins. (**a**,**b**) RAC1. (**c**,**d**) Vinculin. (**e**,**f**) β-actin. (**g**,**h**) Integrin a_v_β_3_ and phalloidin-labeled filamentous actin. The control cells showed the even distribution of a_v_β_3_ integrin (**g**). Type II autosomal dominant osteopetrosis (ADO2) osteoclasts (OCs) showed the uneven distribution of the vitronectin receptor (**h**). The arrows indicate multinuclear cells. a_v_β_3,_ green; actin, red; nuclear, blue.

Several cytoskeleton-related proteins, including RAC1, vinculin, β-actin, and integrin a_v_β_3_, were localized with immunofluorescence to investigate the mechanism underlying the aberrant cellular morphology of ADO2 OCs. No difference was observed in the distribution of RAC1, vinculin, and β-actin between control and ADO2 cells (Figure 6). However, an abnormal distribution of integrin a_v_β_3_ in ADO2 OCs was noted. Specifically, integrin a_v_β_3_ was distributed evenly on the cellular membrane of the control OCs (Figure 6g) but was distributed unevenly on the cellular surface of the ADO2 OCs (Figure 6h).

## Discussion

Our study found enhanced but hypofunctional osteoclastogenesis in an ADO2 subject carrying the c.1856C>T (p.P619L) mutation in exon 20 of *CLCN7*. The PBMCs isolated from this patient showed a strong ability to spontaneously differentiate into OCs. However, the ADO2 OCs that formed presented with extraordinary heterogeneity and aberrant cellular morphology as well as reduced bone resorption capacity.

This report is the first to document a specific germline heterozygous missense mutation, c.1856C>T (p.P619L), in exon 20 of the *CLCN7* gene in ADO2. The case reported here exhibited characteristic bone-in-bone and “sandwich vertebrae” on radiographs, but no overt clinical symptoms of ADO2. A homozygous mutation of c.1856C>T of *CLCN7* was reported previously in an ARO case,^18^ which was diagnosed at 13 months of age. In that case, the patient presented with anemia, optic atrophy, and facial dysmorphism. Previous reports have linked homozygous or compound heterozygous mutations in the *CLCN7* gene to ARO. The majority of ARO cases are secondary to mutations in *TCIRG1*, encoding the a3 subunit of the vacuolar proton pump.^13,19–22^ It has been reported that recessive *CLCN7*-dependent ARO has a very poor prognosis, whereas heterozygous *CLCN7* mutations can lead to a wide range of phenotypes.^22^ Homozygous *CLCN7* mutations are also responsible for an intermediate form of osteopetrosis (IARO), which shows autosomal recessive inheritance, and has a benign prognosis similar to ADO.^21^ In the C terminus of the CLCN7 protein, the region containing Pro619 is highly conserved and is located after helix R.^18^ Our data and those reported by Phadke *et al.*^18^ suggest that this region of the gene is critical for CLCN7 function.

The most prominent characteristic of the ADO2 OCs was their marked heterogeneity. In the present study of ADO2 cultures from PBMCs, only a few cells with normal osteoclast morphology were observed. Instead, giant OCs with large numbers of abnormally arranged nuclei and abnormal TRAP-positive OC-like cells that were aggregated in numerous clumps were common. Others have reported that osteoclasts induced from the bone marrow of a subject with osteosclerosis exhibited heterogeneous morphology.^17^ The heterogeneity of OC morphology and function may explain the variability in the clinical bone phenotypes of ADO2 patients. For example, we speculate that if normal OCs dominate the osteoclast progenitor population, an ADO2 subject may have no obvious clinical symptoms. If, however, aberrant OCs dominate osteoclast progenitors, an ADO2 patient may exhibit an abnormal bone phenotype. Although OCs are derived from hematopoietic cells, their progenitors are not located within a specific or selected subset of macrophages or hematopoietic cell progenitors.^23^ The heterogeneity of OC progenitors may therefore underlie the heterogeneity of OCs and bone phenotypes in ADO2.

Consistent with previous studies, we also found enhanced OC formation in this ADO2 subject. Enhanced OC generation appears to be an intrinsic characteristic of ADO2 because the osteoclast progenitors exhibited abnormally high proliferation and spontaneous differentiation into mature osteoclasts in the absence of OC inducers. The spontaneous generation of osteoclast-like giant cells in bone marrow cultures has also been observed in a patient with osteopetrosis.^11^ Gene analysis found increased expression levels of *c-fos* in ADO2 cells. C-fos, a member of the activator protein-1 (AP-1) family of transcription factors, is involved in regulating the differentiation of OCs.^24^ It is speculated the defective bone resorption of ADO2 OCs leads to increased OC formation, which is reflected by the reduced ratio of Type I collagen C-telopeptide (CTx)/TRAP. The association between increased *c-fos* expression and enhanced osteoclastogenesis in ADO2 needs further investigation.

The detailed mechanism underlying the reduced bone resorption capacity of ADO2 OCs is still unclear. Reduced acid secretion, impaired organic matrix removal, trafficking defects, and a lack of adhesion structures of OCs have been reported to be involved in the defective bone resorption that is observed.^17,25–28^ Our study found that the ADO2 OCs had aberrant cellular morphology; specifically, most were irregularly spindle or satellite-shaped cells, without obvious protrusions and wrinkles. By contrast, the normal OCs were cauliflower-like, with multiple protrusions and wrinkles. When ADO2 osteoclasts were cultured on bone slices, the bone lacunae created by ADO2 OCs were narrow and shallow compared with controls, suggesting a failure of bone resorption. Our data support the hypothesis that in ADO2 osteoclasts, the intracellular re-organization of structural proteins required for osteoclast adhesion to the extracellular matrix may be defective, thus affecting their differentiation, morphology and functionality. Gene analysis of cytoskeleton-related factors found reduced mRNA expression levels of *RhoA*. RhoA regulates actin stress fibers formation and focal adhesions, and the inhibition of RhoA has been found to inhibit actin ring formation and pit formation on bone slices.^29^ As such, the reduced *RhoA* expression in ADO2 OCs may be related to the abnormal adhesion structures of these cells.

In particular, the abnormal expression and distribution of a_v_β_3_ integrin were noted in ADO2 OCs. OCs are rich in a_v_β_3_ integrin, a transmembrane heterodimer that mediates cell/matrix recognition.^30^ Integrin a_v_β_3_ contains an extracellular domain that recognizes bone matrix surface proteins and a cytoplasmic domain that interacts with signaling and cytoskeletal molecules.^30^ The abnormal retention of a_v_β_3_ integrin in cytoplasmic vesicles has been observed in OCs derived from patients with radiographic osteosclerosis.^17,25^ In our study, we also observed an abnormal distribution of a_v_β_3_ integrin in ADO2 OCs compared with controls. In the ADO2 osteoclasts in this study, we observed a down-regulation of *IGTB3* mRNA and the aggregation of a_v_β_3_ integrin in cytoplasm. By contrast, a_v_β_3_ integrin was distributed evenly throughout the cell membrane in control cells. Beta 3 is the regulatory subunit of integrins, and cytokines, including M-CSF and TNF-α, modulate a_v_β_3_ via their effects on β_3_, and not on a_v_, mRNA.^31^ Mice lacking *IGTB3* exhibit an osteosclerotic phenotype, and generate 3.5-fold more osteoclasts with distinctly abnormal cytoskeleton and reduced resorption abilities.^32^ These observations are similar to those observed in our ADO2 subject and in animal models of osteosclerosis.^8^ We speculated that *CLCN7* inactivating mutation may induce downstream effects on the expression and distribution of integrin a_v_β_3_, thereby impairing the cytoskeleton and osteoclastic functions. Because the disruption of a_v_β_3_ integrin appears to be a common feature in osteosclerosis, the correlation between CLCN7 and a_v_β_3_ integrin disruption needs further investigation to determine whether the link between them is one of association or whether it contributes mechanistically to the pathogenesis of osteosclerosis.

*C-fos* is an essential regulator of osteoclastogenesis, and the increased *c-fos* expression observed in this subject may be related to the increase in OC numbers. Elevated *MMP9* expression may also reflect increased OC numbers. CTSK is a critical OC specific lysosomal protease that is involved in bone matrix resorption, and reduced *CTSK* may be related to impaired bone resorption. Abnormal *RhoA* and *IGTB3* expression may also lead to the abnormal cytoskeleton and reduced resorption abilities. However, another study showed the elevated expression of *ITGB5* and reduced expression of *WARS*, *PRF1*, and *SERPINE2* in ADO2 patients. These inconsistent gene profiles may be due to the differences in OC formation and functions.^33^ A previous study suggested that the polymorphisms on the non-disease allele of *CLCN7* and a modifier gene(s) located in chromosome 9q21–22 may affect ADO2 disease status and severity.^34^ The variability in osteoclast functions and clinical phenotypes of ADO2 deserves further investigation.

In conclusion, this study is the first to identify the c.1856C>T (p.P619L) mutation in exon 20 of *CLCN7* gene in an ADO2 subject; this mutation led to enhanced but heterogeneous osteoclastogenesis. Despite the increase in osteoclastogenesis, these giant cells exhibited abnormal morphology and altered functionality *in vitro*. The increased *c-fos* expression in ADO2 OCs may be related to the enhanced osteoclastogenesis observed. The dysregulation of some cytoskeleton-related factors, including RhoA and integrin a_v_β_3_, may be associated with the aberrant cellular morphology and reduced bone resorption of ADO2 OCs.

## Figures and Tables

**Table 1:** Hematopoietic and metabolic data of ADO2 subject

Parameters	ADO2	Reference range
RBC/(10^12^·L^−1^)	5.54	3.8–5.1
Hgb/(g·L^−1^)	171	115–150
WBC/(10^9^·L^−1^)	6.62	4–10
TRAP-5b/(U·L^−1^)	>10	1.3–4.82
CTX/(ng·mL^−1^)	0.364	0.300–0.584
B-ALP/(U·L^−1^)	18.09	11.4–24.6
25-OH-VD/(nmol·L^−1^)	49.50	47.7–144
PTH/(pmol·L^−1^)	6.17	1.60–6.90

Abbrevations: RBC, red blood cells; Hgb, hemoglobin; WBC, white blood cells; TRAP-5b, tartrate-resistant acid phosphatase type 5b; CTX, type I collagen C-telopeptide; B-ALP, bone-specific alkaline phosphatase; PTH, parathyroid hormone.
