# Supplementary figures and images for: Enhanced but hypofunctional osteoclastogenesis in an autosomal dominant osteopetrosis type II case carrying a c.1856C>T mutation in CLCN7
Source: Bone Res. 2016 Nov 29;4:16035–. doi: 10.1038/boneres.2016.35 (PMC5126728; doi:10.1038/boneres.2016.35)

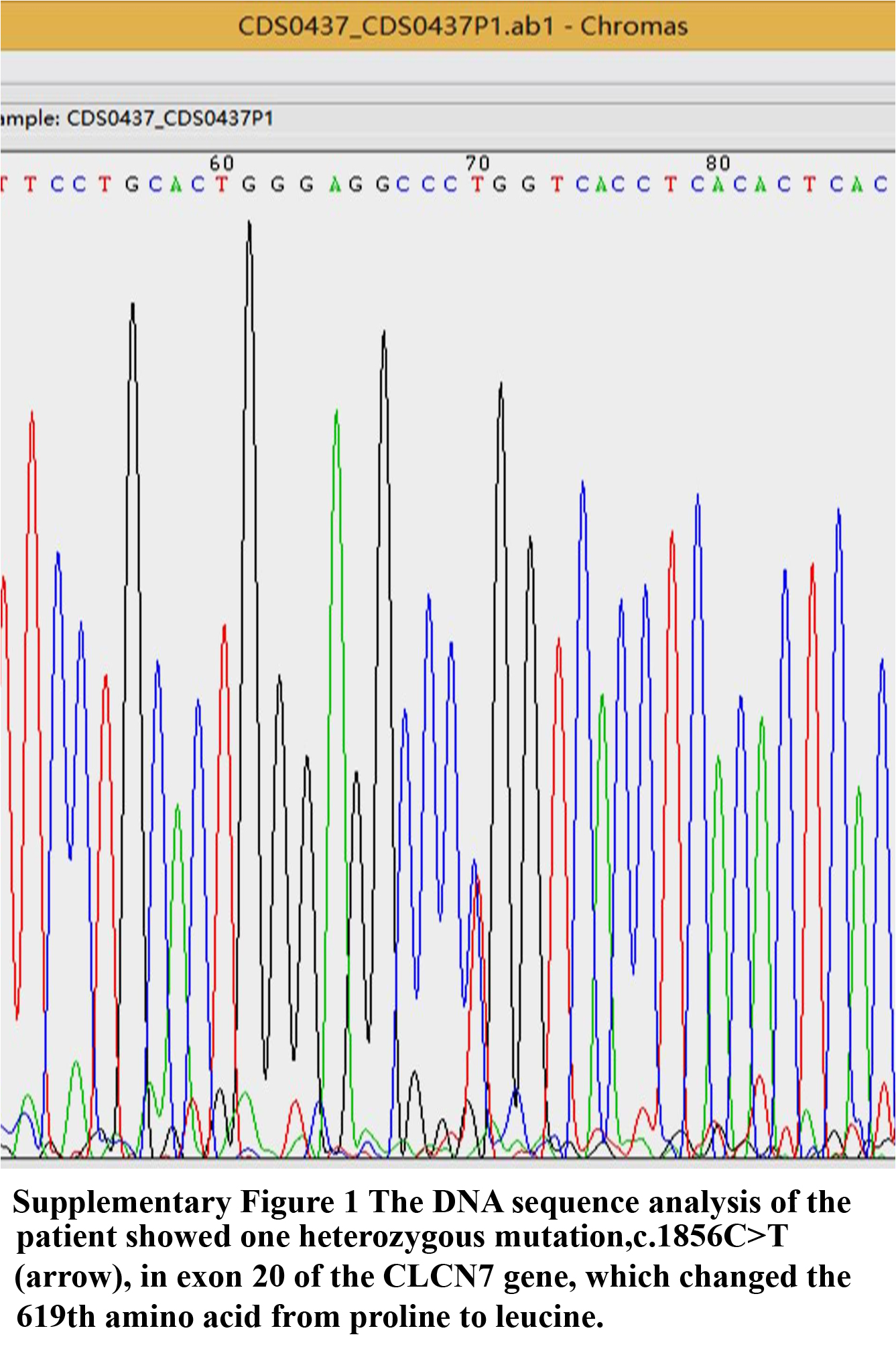

Supplement: Supplementary Figure 1 [file boneres201635-s1.jpg]
